# Inverse Association between Serum Selenium Level and Severity of Liver Fibrosis: A Cross-Sectional Study

**DOI:** 10.3390/nu14173625

**Published:** 2022-09-02

**Authors:** Chi-Wei Shih, Ying-Jen Chen, Wei-Liang Chen

**Affiliations:** 1 Department of Internal Medicine, Tri-Service General Hospital, and School of Medicine, National Defense Medical Center, Taipei 11490, Taiwan; 2 Department of Ophthalmology, Tri-Service General Hospital, and School of Medicine, National Defense Medical Center, Taipei 11490, Taiwan; 3 Division of Family Medicine, Department of Family and Community Medicine, Tri-Service General Hospital, and School of Medicine, National Defense Medical Center, Taipei 11490, Taiwan; 4 Division of Geriatric Medicine, Department of Family and Community Medicine, Tri-Service General Hospital, and School of Medicine, National Defense Medical Center, Taipei 11490, Taiwan

**Keywords:** selenium, trace element, liver fibrosis, transient elastography, NHANES

## Abstract

Selenium has been well recognized for its important role in human health. Prior studies showed that low serum selenium was associated with various diseases, including cardiovascular disease, cancer, infertility, and cognitive decline. Recent studies demonstrated an association between selenium deficiency and liver cirrhosis. In our study, we aimed to explore the association between serum selenium levels and severity of liver fibrosis. In total, 5641 participants at an age of 12 and above, from the 2017–2018 United States National Health and Nutrition Examination Survey, were enrolled. The severity of liver fibrosis was determined by liver ultrasound transient elastography. There was a significant linear decrease in liver stiffness measurement (LSM) values in male groups with increased serum selenium levels. The beta coefficient (β) = −1.045 in male groups. A significantly negative association was also observed in the group of age ≥ 60. In addition, those in the highest quartile of serum selenium had lower LSM values (β = −0.416). This is the first study using LSM to demonstrate the correlation between selenium deficiency and severity of liver cirrhosis. Our findings suggest that a high plasma selenium concentration is negatively correlated with the severity of liver cirrhosis and there are gender and age differences.

## 1. Introduction

As an essential trace mineral, selenium has been well recognized for its important role in human health. Several forms of selenium, such as selenomethionine, selenocysteine, selenite, and selenite, can be found in nature and are absorbed by plants [1]. Once human beings ingest chemical forms of selenium from the diet (selenomethionine, selenocysteine, selenite, and selenite), virtually all of them enter into selenium metabolism and yield selenoproteins, which carry out a wide range of biological functions, including in the musculoskeletal systems, thyroid function, immunity, maintenance of redox homeostasis, and repair of DNA damage [2,3,4]. Prior studies have shown that low serum selenium is associated with various diseases, including cardiovascular disease, liver disease, cancer, infertility, myodegenerative diseases, and cognitive decline [5,6,7,8]. Furthermore, recent epidemiological studies have demonstrated the association between low serum selenium and advanced liver fibrosis [9,10,11].

Liver cirrhosis is the late stage of various chronic liver diseases. The precursor of liver cirrhosis is liver fibrosis, which leads to the accumulation of extracellular matrix proteins such as collagen following liver injury [12]. Although the pathogenesis of liver cirrhosis is multifactorial, oxidative-stress-induced injury in the liver is one of the well-recognized mechanisms [13]. In animal models, selenium supplementation has been shown to improve antioxidant properties and reduce indicative markers of liver cirrhosis [14,15].

Most of the patients are asymptomatic during the progression of liver fibrosis, which makes the diagnosis difficult. Although liver biopsy remains the gold standard of diagnosis, several diagnostic tools and scoring systems have been proposed to achieve an early diagnosis of liver cirrhosis. Mishal Reja et al. demonstrated the correlation between selenium deficiency and severity of liver fibrosis in patients with Nonalcoholic Fatty Liver Disease (NAFLD) by using a NAFLD fibrosis scoring system [9]. In our study, the degree of liver fibrosis was measured by liver ultrasound transient elastography, which is an image test to measure the stiffness of liver tissue. By using a non-invasive and accurate image test, the aim of our study was to explore the association between serum selenium concentration and liver fibrosis.

## 2. Materials and Methods

### 2.1. Study Design and Participants

The National Health and Nutrition Examination Survey (NHANES) is a program of the National Center for Health Statistics (NCHS), which has conducted an annual survey to produce health information and statistics of the US population since 1999. Personal information including demographic information, educational level, medical history, and personal history was obtained by trained examiners during an interview. Physical examination and other medical examinations, including liver ultrasound transient elastography, were conducted in the Mobile Examination Center (MEC). The whole database is released every two years. The NHANES study was approved by the National Center for Health Statistics (NCHS) Institutional Review Board (IRB). The process of informed consent was completed prior to the study.

In the present study, we analyzed the 2017–2018 NHANES datasets. Participants aged 12 years and above were enrolled and underwent liver ultrasound transient elastography. Participants were excluded for the following: (1) missing data, (2) positive serologic markers of hepatitis B or C, (3) transferrin saturation > 50%, (4) aspartate aminotransferase (AST) or alanine aminotransferase (ALT) > 500 IU/L, (5) high alcohol consumption (>20 alcoholic drinks every week for males and >10 alcoholic drinks every week for females), (6) a history of autoimmune hepatitis or primary biliary cirrhosis, (7) pregnancy or inability to provide urine for pregnancy test at the time of the image exam, (7) an electronic medical device implanted in the body, or (8) an injury on the upper right quadrant of the abdomen (where examination took place). These participants were ineligible for the liver ultrasound transient elastography.

### 2.2. Measurement of Trace Elements

Serum levels of selenium, lead, cadmium, and mercury were determined by inductively coupled plasmadynamic reaction cell mass spectrometry (ICP-DRC-MS). The whole blood specimens were collected from participants at Mobile Examination Centers and processed before being shipped to the National Center for Environmental Health in Atlanta, GA for analysis. The details of blood specimen collection, sample preparation, methodology of ICP-DRC-MS, and quality control are discussed in the NHANES Laboratory Procedures Manual [16].

### 2.3. Measurement of Liver Stiffness

The non-invasive quantitation of liver fibrosis was performed by liver ultrasound transient elastography, which is also known as FibroScan^®^. By applying vibration controlled transient elastography (VCTE^TM^), the degree of liver fibrosis can be measured in kilopascals (kPa). Kilopascal values are normally below 6 kPa, and 8 kPa and 12.5 kPa are considered as accepted cut-off values for F3 and F4 fibrosis [17]. In more advanced liver fibrosis, it is more probable to have high portal pressure, and esophageal varices at values of kPa > 20. Compared to the liver biopsy, which is the gold standard for the staging of fibrosis, FibroScan is reported to have high value to assess liver fibrosis and cirrhosis [17,18]. This study acquired FibroScan data from NHANES 2017–2018. The procedure and protocol of FibroScan examination have been well reported in manufacturer guidelines and it was performed by NHANES health technicians in the Mobile Examination Center (MEC) [19]. A complete examination was defined as (1) at least fasting for 3 h, (2) 10 or more complete measurements of liver stiffness (E), and (3) an interquartile (IQRe) range of ≤30% of the median value. Any results that did not meet the definition of complete examination were viewed as partial examination and were not reported, with the exception that if a participant with an E value below the referral criteria had at least 10 complete measurements, the result would be permitted even though the fasting time was not more than 3 h.

### 2.4. Covariates

Demographic information and questionnaire data, such as age, sex, race, use of dietary supplements, and smoking status, were obtained by self-report questionnaires in Mobile Examination Centers. Smoking status was determined by smoking ≥ 100 cigarettes in a lifetime. Use of dietary supplements was determined by the question, ‘Have you ever taken any dietary supplements or vitamins in the past month?’ Biochemistry data, including ALT, total bilirubin, creatinine, and urine albumin, were measured on an automated biochemical analyzer (Cobas 6000 analyzer module c501, Roche). Based on the Beckman Coulter methodology of counting and sizing, platelet counts were obtained by using a Beckman Coulter DxH 800 instrument. Specimen collection and processing procedure details are provided in the NHANES 2017–2018 Laboratory Procedures Manual [16].

### 2.5. Statistical Analyses

The associations between each trace element (selenium, lead, cadmium, and mercury) and liver stiffness were examined using a linear regression model. In addition, we established a model for covariate adjustment, which adjusted for age, sex, BMI and race/ethnicity, platelet count, alanine aminotransferase (ALT), serum creatinine, serum total bilirubin and urine albumin, smoking history, and use of dietary supplements. In addition, we included the Fibrosis (Fib)-4 index in the adjusted model for covariate adjustment. The Fibrosis (Fib)-4 index, which is a non-invasive scoring system for the disease activity of liver fibrosis, is based on several clinical parameters (age, AST, ALT, and platelet count) [20]. Continuous variables and categorical variables were, respectively, assessed by Student’s t test and Pearson’s chi-square tests. Two-sided *p* values of less than or equal to 0.05 were considered statistically significant. In addition, serum selenium levels were divided into quartiles. The cut-off levels for serum selenium quartiles were as follows: Q1 < 2.21 μg/L; Q2: 2.21 to <2.39 μg/L; Q3: 2.39 to <2.60 μg/L; and Q4 >2.60 μg/L. The associations between each quartile and severity of liver stiffness were investigated through the regression model adjusted for multiple covariates. All statistical analyses of this study were performed using the Statistical Package for the Social Sciences, version 18.0 (SPSS Inc., Chicago, IL, USA).

## 3. Results

In total, 5641 participants were enrolled in the study. The mean age was 44.88 ± 20.90 and 49.4% of the participants were men. The mean serum selenium level was 2.41 ± 0.33 μg/L and the mean LSM value was 5.87 ± 5.02 kPa. The clinical characteristics of the study group are shown in Table 1. Since the liver stiffness measurement (LSM) values were associated with serum selenium level (Table 2), we further performed a stratification analysis of subgroups according to sex, age, and BMI, respectively (Table 3). There was a significant linear decrease in LSM values in male groups with increased serum selenium levels in the fully adjusted model, and the regression coefficient was −1.045 (95% CI: −1.746, −0.344; *p* = 0.003). A significantly negative association was also revealed in the subgroup of age ≥60 in the fully adjusted model. In addition, we focused on the correlation between the LSM values and serum selenium level by quantiles. With the weighted population distribution, Table 4 displays the baseline characteristics of the 5641 participants by serum selenium quartile (Q1 < 2.21 μg/L; Q2: 2.21 to < 2.39 μg/L; Q3: 2.39 to < 2.60 μg/L; and Q4 > 2.60 μg/L). Those in the highest quartile of serum selenium (>2.60 μg/L) were older and had significantly higher ALT and total bilirubin but were less likely to have a non-Hispanic black background, compared with the participants in the lowest quartiles. Other demographic, laboratory, and clinical characteristics can be seen in Table 4. Table 5 shows the correlation between the quartiles of serum selenium level and the LSM value. Compared with participants in the lowest quartile, those in the highest serum selenium quartile (>2.60 μg/L) had lower LSM values, as the regression coefficient was −0.416 (95% CI: −0.829, −0.004; *p* < 0.05) after fully multivariate adjustments.

## 4. Discussion

In this nationwide cross-sectional study of the US population, we found an inverse association between the serum level of selenium and severity of liver fibrosis (by LSM value). In the subgroup analysis, the inverse association was shown in individuals with older age. Our result was similar to prior studies that revealed that lower serum selenium was correlated with advanced liver cirrhosis [9,10,21]. In addition, to our knowledge, this is the first study to demonstrate the association between serum selenium and liver cirrhosis with LSM.

In healthy individuals, the serum selenium concentration consists of selenoproteins, selenomethionine, and other forms of serum selenium in an unregulated pool [22]. Selenoprotein P, the most common selenoprotein found in the plasma, is produced by the liver and acts as the main supplier of selenium to extrahepatic tissues. Gpx3 is a functionally characterized selenoprotein. As one of five human glutathione peroxidases (GPx), Gpx3 is produced by the kidneys and is responsible for over 90% of the activity of the GPx in the plasma. The antioxidative properties of selenium and selenoproteins, which are synthesized by the selenium metabolic system, have been recognized. Selenium carries out its antioxidant function via selenoproteins, which protect membrane lipids from the damage of free radicals and reactive oxygen species (ROS) [22]. In addition, in one animal study, one-year-old chicks with selenium supplementation showed significant downregulation of fatty acid synthase and significant upregulation of lipid oxidation enzymes, which protect against liver steatosis [23]. In another animal study of mice with CCl4-induced liver injury and fibrosis, Se-supplemented mice showed decreased stellate cell numbers, which reduced collagen synthesis and enhanced collagen degradation. Thus, Se supplementation slowed the progression of liver fibrosis [24]. As an essential micronutrient for human health, selenium deficiency can contribute to various diseases, including cardiovascular disease, cancer, hepatopathy, and arthropathy [3]. Several studies have proposed the potential role of selenium in reducing hepatic inflammation and fibrosis by suppressing several cytokines, such as TNF-α, IL-6, TGF-β1, and metalloproteinases [24,25,26]. Guo, C.H. et al. conducted a study in which selenium deficiency was associated with elevated levels of oxidative stress markers in the liver, which resulted in liver injury [27]. Dominik Bettinger et al. disclosed a significantly lower serum selenium level in patients with chronic hepatitis C virus infection with liver cirrhosis, compared to patients without liver cirrhosis [10]. Raymond F. Burk et al. revealed a different pattern of serum selenium between patients with liver cirrhosis and healthy individuals with selenium deficiency [20]. Despite a net drop in serum selenium and Selenoprotein P concentration in both groups, glutathione peroxidase activity increased in patients with liver cirrhosis. This might be related to not only a true selenium deficiency but also impairment of selenoprotein synthesis due to hepatic dysfunction. An increase in glutathione peroxidase activity might be a secondary response to cirrhosis. More investigation is needed to elucidate the alteration of selenium metabolism in liver cirrhosis.

The present study carries several clinical implications. Prior studies have shown that selenium supplementation might attenuate the progression of liver disease via its antioxidant activity in animal models [14,21]. In our study, the supplementation of selenium might have exerted possible hepato-protective effects on individuals aged over 60 years who had liver cirrhosis. On the other hand, serum selenium, as an independent correlate, shall be considered as a confounder for individuals with liver cirrhosis in future studies. The different impacts of selenium on different genders requires further studies for elucidation.

There were several limitations in our study. First, owing to the study design, the protective effect of selenium could not be established in this cross-sectional study. Hence, more prospective studies and randomized controlled trials are needed before making a recommendation regarding selenium supplementation. Second, our study provided an insightful observation about the correlation between selenium deficiency and the severity of liver cirrhosis; however, a liver biopsy remains the gold standard for diagnosing liver cirrhosis. Third, because of racial differences in serum selenium concentration, more research is required to confirm the recommended level of serum selenium for each race [28].

## 5. Conclusions

In conclusion, this is the first study to demonstrate the correlation between selenium deficiency and severity of liver cirrhosis by using LSM. The serum selenium level is inversely associated with the severity of liver cirrhosis. Although further research is necessary to explain the causality among serum selenium levels and liver cirrhosis, our findings suggest that selenium plays an important role in liver fibrosis and indicate gender differences in the association. Further longitudinal research with an interventional design is necessary to confirm the conclusions.

## Figures and Tables

**Table 1:** Characteristics of study participants.

Continuous Variables			
	Male (*n* = 2784)	Female (*n* = 2857)	*p*
Age (years)	44.75 (21.24)	45.01 (20.59)	0.004
BMI	28.38 (6.70)	29.33 (8.04)	<0.001
Creatinine (mg/dL)	0.10 (0.52)	0.75 (0.31)	<0.001
ALT(U/L)	25.32 (18.11)	17.88 (15.04)	<0.001
Total bilirubin (mg/dL)	0.53 (0.31)	0.40 (0.24)	<0.001
Platelet count (1000 cells/uL)	231.07 (57.14)	262.31 (65.67)	<0.001
Blood selenium (umol/L)	2.44 (0.33)	2.40 (0.33)	0.707
Blood lead (umol/L)	0.06 (0.08)	0.05 (0.04)	<0.001
Blood cadmium (umol/L)	3.48 (4.15)	4.21 (5.35)	0.002
Blood mercury	6.60 (12.98)	6.56 (12.16)	0.786
Blood manganese	171.61 (59.15)	196.73 (74.18)	<0.001
Albumin, urine (ug/mL)	47.06 (316.42)	50.49 (341.18)	0.385
Median stiffness (kPa)	6.29 (5.56)	5.47 (4.41)	<0.001
FIB-4 index	1.07 (1.09)	0.93 (0.70)	<0.001
Categorical variables	male	female	*p*
Race (%)			0.34
Non-Hispanic white	34.4	33.2	
Non-Hispanic black	22.1	23.0	
Mexican American	14.7	14.8	
Other Hispanic	8.7	9.7	
Other race	20.2	19.0	
Smoked (%)	50.5	30.8	<0.001
Use of dietary supplements	44.3	57.2	<0.001

Continuous variables are presented as mean (standard deviation). Categorical variables are presented as percentage. Abbreviations: CRP = C-reactive protein, ALT = alanine aminotransferase, kPa = kilopascals, BMI = body mass index, FIB-4 index = Fibrosis-4 index.

**Table 2:** Associations between circulating concentrations of trace elements and liver stiffness.

	Unadjusted *β* (95% CI)		Fully Adjusted *β* (95% CI)	
Trace Elements	*β* (95% CI)	*p*	*β* (95% CI)	*p*
Lead (μmol/L)	0.538	0.659	−0.496	0.68
	(−1.855, 2.931)		(−2.851, 1.859)	
Cadmium (μmol/L)	−0.029	0.059	−0.018	0.261
	(−0.06, 0.001)		(−0.049, 0.013)	
Mercury (μmol/L)	−0.013	0.028	−0.007	0.218
	(−0.024, −0.001)		(−0.018, 0.004)	
Selenium (μmol/L)	−0.548	0.02	−0.721	0.001
	(−1.008, −0.087)		(−1.157, −0.285)	
Manganese (μmol/L)	0.001	0.573	0.001	0.196
	(−0.002, 0.003)		(−0.001, 0.004)	

Adjusted covariates: fully adjusted model: age + gender + race + BMI + platelet count + urine albumin + ALT + serum creatinine + serum total bilirubin + cigarette smoking + FIB-4 index + use of dietary supplements.

**Table 3:** Associations between serum selenium concentration and liver stiffness by gender, age, and BMI.

	Unadjusted	Fully Adjusted		
	*β* (95% CI)	*p*	*β* (95% CI)	*p*
Male (*n* = 2784)	−0.818 (−1.550, −0.086)	0.029	−1.045 (−1.746, −0.344)	0.003
Female (*n* = 2857)	−0.488 (−1.054, 0.078)	0.091	−0.385 (−0.913, 0.143)	0.153
Age < 60 (*n* = 4013)	0.001 (−0.582, 0.583)	0.998	−0.078 (−0.614, 0.458)	0.777
Age ≥ 60 (*n* = 1628)	−1.273 (−2.030, −0.515)	0.001	−1.315 (−2.054, −0.575)	<0.001
BMI < 30 (*n* = 3522)	−0.257 (−0.657, 0.144)	0.209	−0.383 (−0.780, 0.014)	0.059
BMI ≥ 30 (*n* = 2072)	−0.930 (−1.907, 0.047)	0.062	−0.743 (−1.646, 0.160)	0.107

Adjusted covariates: fully adjusted model: age + gender + race + BMI + platelet count + urine albumin + ALT + serum creatinine + serum total bilirubin + cigarette smoking + FIB-4 index + use of dietary supplements.

**Table 4:** Characteristics of study participants by quartile of serum selenium concentration (Quartiles 1–4).

	Quartiles of Serum Selenium Concentration (µg/L)					
Characteristics	Q1 (<2.21)	Q2 (2.21 to <2.39)	Q3 (2.39 to <2.60)	Q4 (>2.60)	Total	*p*
	(*n* = 1413)	(*n* = 1407)	(*n* = 1411)	(*n* = 1410)	(*n* = 5641)	Value
Age (years)	44.22 (22.30)	44.43 (20.84)	44.39 (20.29)	46.49 (20.06)	44.88 (20.91)	0.011
Sex (% male)	688 (48.7%)	712 (50.6%)	684 (48.5%)	700 (49.6%)	2784 (49.4%)	0.657
Race (%)						<0.001
Non-Hispanic white	474 (33.5%)	437 (31.1%)	495 (35.1%)	502 (35.6%)	1908 (33.8%)	
Non-Hispanic black	357 (25.3%)	344 (24.4%)	300 (21.3%)	270 (19.1%)	1271 (22.5%)	
Mexican American	192 (13.6%)	227 (16.1%)	220 (15.6%)	193 (13.7%)	832 (14.7%)	
Other Hispanic	134 (9.5%)	140 (10.0%)	120 (8.5%)	131 (9.3%)	525 (9.3%)	
Other race	256 (18.1%)	259 (18.4%)	276 (19.6%)	314 (22.3%)	1105 (19.6%)	
BMI	28.37 (7.70)	28.78 (7.57)	29.03 (7.25)	29.26 (7.15)	28.86 (7.42)	0.012
Creatinine(mg/dL)	0.92 (0.68)	0.86 (0.38)	0.85 (0.27)	0.86 (0.30)	0.87 (0.44)	<0.001
ALT(U/L)	19.48 (14.57)	20.93 (15.68)	21.60 (15.53)	24.19 (21.20)	21.55 (17.03)	<0.001
Total bilirubin (mg/dL)	0.44 (0.27)	0.44 (0.26)	0.47 (0.28)	0.50 (0.31)	0.46 (0.28)	<0.001
Platelet count (1000 cells/uL)	244.04 (66.83)	248.08 (64.02)	250.05 (63.85)	245.38 (59.15)	246.89 (63.54)	0.055
Blood selenium (umol/L)	2.05 (0.14)	2.30 (0.06)	2.49 (0.06)	2.83 (0.29)	2.42 (0.33)	<0.001
Albumin, urine (ug/mL)	72.38 (557.21)	43.43 (228.94)	37.28 (217.75)	42.41 (158.70)	48.80 (329.21)	0.021
Median stiffness (kPa)	6.06 (5.49)	5.81 (5.22)	5.77 (4.73)	5.87 (4.62)	5.88 (5.03)	0.434
FIB-4 index	1.08 (0.95)	0.95 (0.68)	0.96 (1.21)	0.99 (0.72)	1.00 (0.92)	0.001
Smoked (%)	518 (44.1%)	486 (39.9%)	466 (38.3%)	504 (39.8%)	1974 (40.5%)	0.026
Use of dietary supplements	658 (46.6%)	686 (48.8%)	742 (52.6%)	782 (55.5%)	2868 (50.8%)	<0.001

Continuous variables are presented as mean (standard deviation). Categorical variables are presented as percentage.

**Table 5:** Associations between serum selenium concentration and liver stiffness by quartile of serum selenium concentration (Quartiles 1–4).

	Unadjusted		Fully Adjusted	
	*β* (95% CI)	*p*	*β* (95% CI)	*p*
Quartile 1	1.0 (reference)		1.0 (reference)	
Quartile 2	−0.290 (−0.732, 0.151)	0.198	−0.241 (−0.655, 0.172)	0.252
Quartile 3	−0.344 (−0.785, 0.098)	0.127	−0.378 (−0.792, 0.037)	0.074
Quartile 4	−0.300 (−0.737, 0.137)	0.178	−0.416 (−0.829, −0.004)	<0.05

Adjusted covariates: fully adjusted model: age + gender + race + BMI + platelet count + urine albumin + ALT + serum creatinine + serum total bilirubin + cigarette smoking + FIB-4 index + use of dietary supplements.

## Data Availability

The data presented in this study are available on request from the corresponding author.
